# Genome-Wide Immune Modulation of TLR3-Mediated Inflammation in Intestinal Epithelial Cells Differs between Single and Multi-Strain Probiotic Combination

**DOI:** 10.1371/journal.pone.0169847

**Published:** 2017-01-18

**Authors:** Chad W. MacPherson, Padmaja Shastri, Olivier Mathieu, Thomas A. Tompkins, Pierre Burguière

**Affiliations:** 1 Lallemand Health Solutions Inc., 6100 avenue Royalmount, Montreal, QC, Canada; 2 University of Ontario Institute of Technology, Oshawa, Canada; Duke University, UNITED STATES

## Abstract

Genome-wide transcriptional analysis in intestinal epithelial cells (IEC) can aid in elucidating the impact of single versus multi-strain probiotic combinations on immunological and cellular mechanisms of action. In this study we used human expression microarray chips in an *in vitro* intestinal epithelial cell model to investigate the impact of three probiotic bacteria, *Lactobacillus helveticus* R0052 (Lh-R0052), *Bifidobacterium longum* subsp. *infantis* R0033 (Bl-R0033) and *Bifidobacterium bifidum* R0071 (Bb-R0071) individually and in combination, and of a surface-layer protein (SLP) purified from Lh-R0052, on HT-29 cells’ transcriptional profile to poly(I:C)-induced inflammation. Hierarchical heat map clustering, Set Distiller and String analyses revealed that the effects of Lh-R0052 and Bb-R0071 diverged from those of Bl-R0033 and Lh-R0052-SLP. It was evident from the global analyses with respect to the immune, cellular and homeostasis related pathways that the co-challenge with probiotic combination (PC) vastly differed in its effect from the single strains and Lh-R0052-SLP treatments. The multi-strain PC resulted in a greater reduction of modulated genes, found through functional connections between immune and cellular pathways. Cytokine and chemokine analyses based on specific outcomes from the TNF-α and NF-κB signaling pathways revealed single, multi-strain and Lh-R0052-SLP specific attenuation of the majority of proteins measured (*TNF-α*, *IL-8*, *CXCL1*, *CXCL2* and *CXCL10*), indicating potentially different mechanisms. These findings indicate a synergistic effect of the bacterial combinations relative to the single strain and Lh-R0052-SLP treatments in resolving toll-like receptor 3 (TLR3)-induced inflammation in IEC and maintaining cellular homeostasis, reinforcing the rationale for using multi-strain formulations as a probiotic.

## Introduction

In the past decade a number of studies have established important beneficial effects of probiotics that include alteration of gut microbiota, competitive adherence to gut epithelium, enhancement of the intestinal epithelial barrier function and immune modulation of various cell types of the gut-associated lymphoid tissue (GALT) [1,2]. One of the core properties of probiotic bacteria is their capacity to modulate innate and adaptive immune responses of different cell types of the GALT that include intestinal epithelial cells (IEC), mast cells (M cells), dendritic cells (DC), macrophages, T-cells and B-cells [3,4]. Several key immune pathways such as Jak/STAT, mitogen-activated protein kinase (MAPK) and nuclear factor kappa-light-chain-enhancer of activated B cells (NF-κB) signaling cascades have been implicated as possible sources behind the mechanism(s) of action of probiotics [5,6]. However, the elucidation of the exact mechanism(s) underlying how probiotic bacteria direct their beneficial effects on the host are not entirely understood [7].

Interestingly, studies have also established strain specific effects of probiotic bacteria on immune modulation and barrier function [8,9]. Moreover, a few studies that have compared single strains to multi-strain combinations have revealed, in some cases synergistic and in other situations antagonist, effects [9,10]. Preclinical assessment of blood samples from two distinct rat models of infection (T_H_1 and T_H_2) using single strains of Lh-R0052, Bl-R0033 and Bb-R0071 and the multi-strain probiotic combination of all 3 reported that the multi-strain synergistically benefited both T_H_1 and T_H_2 responses, but the mechanism(s) and the contribution of each strain was not elucidated [11].

Components of bacteria such as surface layer proteins (SLP) also influence several mechanisms including adhesion and are expressed by several probiotics including lactobacilli [12]. The receptor-binding region of the SLP from *L*. *brevis* adheres to gut epithelial cells, is transferrable to other non-adhesive LAB [13] and can also bind to blood type A-antigen found in the gut, aiding in intestinal colonization in humans [14]. In addition, *in vitro* application of SLP extracted from *L*. *helveticus* to epithelial cells (Hep-2 and T84) helps decrease adherence of *E*. *coli* O157:H7 [15]

Evidence from various studies suggests probiotics and SLP interact with pattern recognition receptors (PRRs) that include Toll-like receptors(TLR), nucleotide-binding oligomerization-like receptors (NOD-like receptors), adhesion molecules and lectins, in turn directing the modulation of key signaling pathways such as MAPK and NF-κB [1,4,16]. Studies of immune-modulation of TLR-mediated responses have mainly focused on the effects of different strains of *Lactobacillus rhamnosus* and include the differential expression of TLR genes in human primary macrophages [17], polarization of human monocyte-derived dendritic cells via TLR2 [18] and the attenuation of TLR4-induced signaling in *Escherichia coli-*challenged immune cells [19,20]. *L*. *helveticus* SLP acting through TLR2 in macrophages stimulates a pro-inflammatory response, while dampening NF-κB activation in intestinal epithelial cells [16], illustrating a different effect by bacterial components compared to whole bacteria at an immunological level on different cell types.

Relatively few studies have examined the effect probiotic strains have on TLR3-mediated immune responses. While most TLR primarily act through the MYD88-dependent pathway, TLR3, which detects dsRNA, triggers the MYD88-independent pathway. TLR3 activation does not involve the Toll/Interleukin 1-MYD88 interaction, but instead utilizes the adaptor protein TRIF (Toll/Interleukin 1 domain containing adaptor inducing IFN-β),-leading to IRF3-mediated transcriptional events. The MYD88-independent pathway also leads to the transcription of IFN-βinducible genes such as *CXCL10* [21]. This difference in signaling from other TLR makes TLR3 an attractive PRR through which to study probiotic activity.

We have previously demonstrated through a custom-designed immune microarray analysis a multi-strain (Bl-R0033, Lh-R0052 and Bb-R0071) probiotic combination (PC) on TLR3-induced immune activation by polyinosinic:polycytidylic acid (poly(I:C)) in human intestinal epithelial cells attenuated T_H_1 pro-inflammatory response through the TLR3-TRIF, MAPK and NF-_K_B signaling pathways [22]. However, recent evidence also indicates a single probiotic strain, *L*. *rhamnosus* GG, can increase the expression of TLR3, specifically, in murine intestinal organoids [23], indicating a potential difference between single versus multi-strain probiotic combination on TLR3-related immune activity at the intestinal epithelial level.

The aim of this study was to elucidate the impact of the probiotic bacterial strains (Bl-R0033, Lh-R0052 and Bb-R0071) individually and in combination, and a specific surface-layer protein purified from Lh-R0052 (Lh-R0052-SLP), in response to TLR3-engagement in an *in vitro* IEC model. Genome-wide human expression microarrays were utilized to evaluate other cellular pathways beyond the immune-related pathways, especially in light of the benefits associated with probiotics on nervous, endocrine, stress-related behavior [24] and various gut inflammatory or autoimmune disorders [25,26].

## Materials and Methods

### Intestinal Epithelial Cell Culture

Human colon adenocarcinoma (HT-29) cells were purchased from American Type Culture Collection (ATCC #HTB-38, Cedarlane, Canada), and cultured in a suspension of RPMI-1640 media (HyClone, Logan, UT, USA) supplemented with 5% bovine calf serum, 5% fetal bovine serum and 2 mM L-glutamine (Invitrogen, Life Technologies). Cell cultures were grown in T75-cm^2^ tissue culture flasks (Corning Life Sciences, Acton, MA) at 37°C in a humidified, 5% CO_2_ incubator (Steri-cycle, ThermoFisher Scientific). Cultures were routinely passaged when they reached a confluence ~75–90% and used for subsequent challenge experiments between passages 8–22. For all challenge experiments, HT-29 cells were seeded at 2.5 x 10^6^ cells and grown for 48 h in standard tissue-culture T25-cm^2^ flasks to reach a final total cell count of ~5 x 10^6^ cells. Cells were washed with Dulbecco’s Phosphate Buffered Saline (DPBS) (HyClone, Logan, UT, USA) and incubated 30 min in serum-free RPMI prior to challenge assays.

### Bacteria and Culture Conditions

Laboratory blend of the multi-strain bacteria referred to as multi-strain or probiotic combination, PC, was prepared using industrially prepared lyophilized bacterial powders (Lallemand Health Solutions Inc., Montreal, QC, Canada) of *B*. *longum* subsp. *infantis* R0033, *B*. *bifidum* R0071 and *L*. *helveticus* R0052 in a ratio of 20:20:60; respectively, and as described in MacPherson *et al*. [22]. To rehydrate the lyophilized bacteria for both single and multi-strain blend, 1 g was mixed for 15 min at room temperature (RT) in 99 mL of phosphate buffer [0.1% soy peptone (w/v), 0.121% K_2_HPO_4_ (w/v), 0.034% KH_2_PO_4_ (w/v)] as described in MacPherson *et al*. and Audy *et al*. [22,27]. Briefly, bacterial pellet from 1 ml of this bacterial suspension was washed in PBS after centrifugation at 12800 x g for 10 min at room temperature (RT) and then re-suspended in serum-free RPMI-1640 media. Individual bacteria and the multi-strain probiotic combination (PC) suspension was added to the culture flask (T25-cm^2^ flasks containing HT-29 cells) to have a multiplicity of infection (MOI) of 100:1 for bacteria to HT-29 cell ratio. Viable counts using reinforced clostridial agar (Oxoid) were performed on the bacterial suspension and incubated 48 h anaerobically at 37°C to confirm the calculated ratio.

### Surface Layer Protein Extraction from L. helveticus R0052

Extraction of surface-layer protein from Lh-R0052 was performed with LiCl based extraction method as described in Johnson-Henry *et al* [15] and Taverniti *et al* [16]. Briefly, Lh-R0052 culture was grown overnight (16~18 hours) in anaerobic jars in 9 mL of MRS broth at 37°C. Overnight culture was transferred to 500 mL MRS broth and again incubated overnight in the same conditions. Bacterial culture (500 ml) was harvested in 50 mL Falcon tubes by centrifugation at 7,000 rpm for 10 min at 4°C, washed once with 1 volume of cold sterile distilled H_2_O and centrifuged again in same conditions. Bacterial cell pellet was re-suspended with 1M LiCl solution, incubated for 30 min at RT in the presence of protease inhibitor cocktail (0.001%; Sigma-Aldrich, St. Louis, MO) with slight agitation and centrifuged at 10,000 x g for 20 minutes at RT. The supernatant was discarded and the cell pellet was extracted for surface-layer protein with 5M LiCl solution for 1h at RT in the presence of protease inhibitor cocktail (0.001%) and centrifuged at 10,000 x g for 20 minutes at RT. The supernatant (containing surface-layer protein) was filtered through a 0.22 μm-pore-size filter and dialyzed for 24 h at 4°C against distilled H_2_O using 12 kDa cutoff membranes (Sigma-Aldrich). Dialysis tubing was prepared by boiling for 10 min in 2% NaHCO_3_ and 1mM EDTA solution. Dialysis tubing of ~20 cm in length was filled with ~20 mL of collected protein extract supernatant to dialyze sample in 4 L beaker filled with sterile distilled H_2_O to remove 5M LiCl at 4°C with periodic changing of the water. At each water change, 0.001% protease inhibitor cocktail was added. Once the dialysis was completed the samples were collected from the tubing and freeze-dried. Samples were stored at -20°C until later use. Protein concentration of extracted surface-layer protein from Lh-R0052 was determined by measuring on the Direct Detect® (EMD Millipore, Billerica, MA) spectrometer.

### 1D-SDS-PAGE and 2D-gel Analyses

LiCl extracted Lh-R0052-SLP preparations were assessed for correct molecular weight by running protein preparations on 15% SDS-PAGE precast Mini-Protean TGX gels (Bio-Rad; Cat. 456–1083). Briefly, 2, 4, and 8 μg of Lh-R0052-SLP preparation were added to 1X SDS loading dye, and incubated at 100°C for 10 min before loading on gel. SDS-PAGE gel was run for 1.5 hr at 120 volts, stained overnight with Bio-Safe^TM^ Coomassie G-250 stain (Bio-Rad; Cat. 101–0786) and destained with distilled water. Aliquoted samples of Lh-R0052-SLP were also sent to Applied Biomics (www.appliedbiomics.com/) for 2D-difference gel electrophoresis (DIGE) to determine the isoelectric point and purity of the Lh-R0052-SLP. Both 1D SDS-PAGE and 2D-gel analysis showed that the Lh- R0052-SLP migrated to the expected molecular weight and isoelectric point of 48 kDa and 9.34; respectively, (S1 Fig) and was consistent with what was reported in Johnson-Henry *et al*. [15]. The purity of the LiCl extracted Lh-R0052-SLP preparation was further assessed on 2D-gel by probing with rabbit polyclonal antibody specific for Lh-R0052-SLP (available from Lallemand Health Solutions). The protein purity was calculated to be 99.12%. The equivalent amount of surface layer protein to be used in challenges with IEC was also determined by loading a known amount of bacterial cells onto 2D-gel and probing with the same rabbit polyclonal antibody that is specific for Lh-R0052-SLP (available from Lallemand Health Solutions Inc., Montreal). An equivalent amount of Lh-R0052-SLP protein based on 5 x 10E8 bacteria cells to have an equivalent MOI 100:1 was calculated to be 38μg/ml in a 5 ml culture of HT-29 cells.

### HT-29 Cell Challenges

Cell challenges were performed as explained previously in MacPherson *et al*. and Audy *et al*. [22,27]. Briefly, HT-29 cells were co-challenged for 3h with either the single strains (Bl-R0033, Lh-R0052 or Bb-R0071) or probiotic combination (PC) alone or in combination with poly(I:C) at 10 μg/mL (Sigma; Cat. P1530). Lh-R0052-SLP challenges used alone or in combination with poly(I:C) were performed as previously described [22]. Challenges for cytokine and chemokine profiling were performed over 6h to allow for the accumulation of protein levels as described previously [27].

### RNA Extraction and Dye-labeling

Total RNA isolation was performed on HT-29 cells using a phenol-based extraction method as described previously [22,27]. 15 μg of control and treated RNA was used for reverse transcription to cDNA and direct method of dye-labeling using Cy3-dCTP and Cy5-dCTP as described in MacPherson *et al*. [22].

### Hybridization and Scanning

Genome-wide human expression microarrays version 2 were purchased from Agilent Technologies Inc. (GE 4x44K; G2519F). Briefly, Agilent array pre-hybridization, hybridization and post-hybridization were performed as previously described [22]. After post-hybridization, slides were scanned using ScanArray 5000 instrument from Perkin-Elmer (Waltham, MA) and spot intensities were quantified using ImaGene® version 9.0 (BioDiscovery).

### Microarray Statistical Analysis

Global LOWESS normalization was conducted with ImaGene® microarray analysis software. Statistical analyses and two-dimensional hierarchical clustering analyses were performed with Multi-Experiment Viewer (MeV, version 4.9), a freely available bioinformatics analyses tool of the TM4 microarray software suite from the J. Craig Venter Institute [28]. Genes with changes in transcript abundance were selected on the basis of two criteria: (i) a *t*-test *p-*value of less than 0.05, which was considered statistically significant, and (ii) a cut-off in transcript abundance of least 1.5-fold change. Information regarding the microarray platform and the expression data files can be found on the NCBI Gene Expression Omnibus (GEO; http://www.ncbi.nlm.nih.gov/geo/) under GEO platform no. GPL10332 and GEO series no. GSE71515.

### Set Distiller and String Analyses

Enrichment analyses of the genes modulated by each challenge or co-challenges were done using Set Distiller from GeneDecks version 3 [29] which ranks gene sets into attribute types (e.g., super pathways, KEGG pathways, compounds, phenotypes, expression and disorders) and descriptors (pathways) that best characterize the entire gene set. A *p*-value <0.05 was considered to be statistically significant for enrichment of pathways in Set Distiller (Bonferroni corrected). Interaction network maps were constructed using String version 9.1 [30] for selected pathways and disorders from the Set Distiller analyses to determine functional links between genes, verify the Set Distiller analyses and discover new relevant biological insights that had been overlooked.

### Cytokine and Chemokine Profiling

Bio-Plex Pro^TM^ human cytokine and chemokine standards were serially diluted for the establishment of calibration curves for the determination of protein concentration as per the manufacturer’s instructions (Bio-Rad, Richmond, CA). All experiments were conducted with 4 biological replicates with 2 technical replications per biological replicate. All cytokine and chemokine measurements (*TNF-α*, *IL-1β*, *IL-6*, *IL-8*, *CXCL1*, *CXCL2* and *CXCL10*) were multiplexed in the same 96-well plate. Bio-Plex Pro^TM^ software was used to determine the protein concentration using concentration in range and represented in pg/ml. Results were presented as the means ± SD of the replicate experiments. One-way ANOVA using Dunnett’s multiple comparisons test was performed with GraphPad version 6 to determine the statistical significance with the poly(I:C)-only challenge, compared to the co-challenges of Bl-R0033, Lh-R0052, Bb-R0071, Lh-R0052-SLP and PC. *P-value* <0.05 was considered to be statistically significant for each of the co-challenges and controls compared to the poly(I:C)-only challenge.

## Results

### Heat-map Analyses

**Fig 1:**
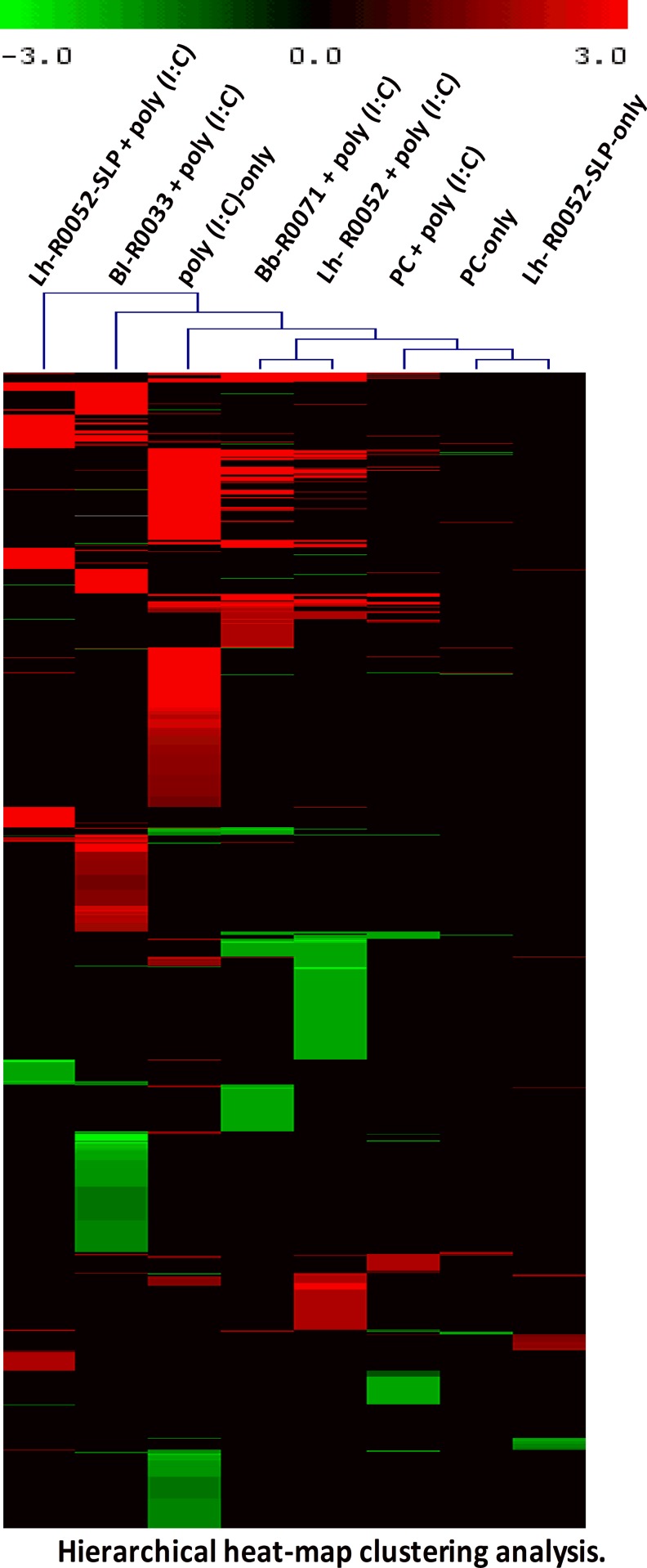
Two-dimensional hierarchical heat-map clustering analysis showing differentially modulated genes for each challenge and co-challenge of HT-29 cells. Genes included in the heat map analysis were statistically significant with a *p*-value of <0.05 and a cut-off of 1.5-fold change in differential gene expression. Genes that were up-regulated are shown in red and down-regulated in green.

Genome-wide transcriptional analysis was used to investigate the transcriptional response of HT-29 cells co-challenged with individual bacterial strains (Bl-R0033, Lh-R0052 and Bb-R0071), probiotic combination (PC) and a purified Lh-R0052-SLP in the presence of the TLR3 inducer poly(I:C). Two-dimensional hierarchical clustering grouped individual challenges and differentially modulated genes into a clustering tree with similar expression profiles as depicted in Fig 1. The heat map analysis revealed a number of global observations from each of the challenges and co-challenges (Fig 1). The poly(I:C)-only challenge had the greatest impact on differential gene expression by modulating 607 genes, whereas the co-challenge with Bl-R0033, Lh-R0052, Bb-R0071, Lh-R0052-SLP or PC with poly(I:C) had a lower impact of modulating 467, 368, 293, 219 and 131 genes respectively (Table 1). The results indicated that the large number of genes that were differentially modulated by the poly(I:C)-only challenge were either attenuated or turned off by the probiotic co-challenges.

**Table 1:** Total number of up-regulated and down-regulated genes for each challenge and co-challenge. All genes modulation are statistically significant with a p-value <0.05 and a cut-off of transcript abundance of 1.5-fold.

	Number of Genes Modulated		
Challenges/Co-challenges	Up-regulated	Down-regulated	Total
poly(I:C)-only	469	138	607
Lh-R0052-SLP + poly(I:C)	175	44	219
Bl-R0033 + poly(I:C)	268	199	467
Bb-R0071 + poly(I:C)	170	123	293
Lh-R0052 + poly(I:C)	167	200	367
PC + poly(I:C)	60	71	131
PC-only	7	10	17
R0052-SLP-only	30	18	48

The individual bacterial co-challenges had strain specific effects on gene modulation. Each single strain induced unique gene modulation not observed with the other co-challenges (Fig 1). Co-challenge with Lh-R0052-SLP also had a unique impact on gene modulation compared to the other co-challenges. Although each of the individual bacteria and Lh-R0052-SLP had a positive effect on attenuating global gene expression, the multi-strain PC had the greatest impact. The PC reduced the number of modulated genes from 607 to 131 genes (Table 1). Without prior poly(I:C) stimulation, the PC-only and Lh-R0052-SLP-only challenges modulated only 17 and 47 genes; respectively.

### Set Distiller Enrichment Analysis

In order to have a better understanding of what pathways were modulated and what genes were implicated, enrichment analysis was performed using the bioinformatics tool Set Distiller from GeneDeck. As previously explained, Set Distiller takes gene sets and categorizes descriptors (pathways) that best characterize a particular gene set [29]. The results of the enrichment analyses for each of the challenges were placed into pathways that corresponded to immune, cellular signaling, compound, virus, endocrine/nervous and disorder related (Tables 2 and 3). The enrichment analyses illustrated that there were numerous pathways associated with cellular signaling. Notable pathways related to cellular signaling included homeostasis/metabolism, PAK pathway, focal adhesion, and apoptosis signaling pathway (Table 2). Overall, results revealed that the poly(I:C)-only challenge had a greater impact on this model system, as evident by the larger number of pathways and the total number of genes modulated. For example, the poly(I:C)-only challenge exhibited a greater impact on gene modulation for immune related pathways such as TNF-α (16), Jak-STAT (7), MAPK (21), NF-κB family pathway (17), cytokine-cytokine receptor interaction (16) and chemokine (13), Immune response IL23 (12), IL-17 family (9) and Toll-like receptor (8) signaling pathways. Examples of pro-inflammatory genes that were up-regulated by poly (I:C)-only in these immune pathways included *TNF-α* (32.4-fold), *RELB* (11.3-fold), *IL-8* (7.2-fold), *CXCL1*, (5.7-fold) *CXCL2* (4.6-fold), *CXCL3* (4.2-fold), *CXCL10* (2.6-fold) and *LIF* (2.0-fold) (S1 Table). The up-regulation of these genes confirmed previously reported data using a customized Immune Array [22]. Furthermore, there was a progressive reduction in the total number of genes for a number of pathways for the single strains and Lh-R0052-SLP; with the multi-strain PC showing the greatest impact at reducing the number of genes induced by poly(I:C)-only (Tables 2 and 3; S1 Table).

**Table 2:** Enrichment analysis using Set Distiller analysis from GeneDecks (version 3) showing descriptors/pathways and the number of genes modulated for immune, compound and cellular related pathways. Enrichment analysis of pathways are statistically significant with a p-value <0.05 (Bonferroni corrected).

GeneDecks Set Distiller Analysis	Attribute Type	poly(I:C)	Lh-R0052	Bb-R0071	Bl-R0033	Lh-R0052-SLP	PC	PC	Lh-R0052-SLP
		only	poly(I:C)	poly(I:C)	poly(I:C)	poly (I:C)	poly(I:C)	only	only
**Immune Related**	**Number of Genes Modulated**								
Immune System Phenotype	PHENOTYPE	88	85	77	59	32	22	-	-
TNF Signaling Pathway	KEGG_PATHWAY	16	12	14	-	-	-	-	-
MAPK Signaling Pathway	SUPER_PATHWAY	21	16	14	6	-	4	-	-
NF-KappaB Signaling Pathway	KEGG_PATHWAY	13	14	12	4	3	7	-	-
NF-KappaB Family Pathway	SUPER_PATHWAY	17	13	11	-	-	1	-	-
Jak-STAT Signaling Pathway	SUPER_PATHWAY	7	1	2	-	3	1	-	-
Immune response IFN Alpha/Beta Signaling Pathway	SUPER_PATHWAY	8	-	-	-	-	-	-	-
Cytokine-cytokine Receptor Interaction	KEGG _PATHWAY	16	16	12	8	7	7	-	-
Chemokine Signaling	KEGG_PATHWAY	13	14	6	-	6	6	-	-
Toll-like receptor Signaling Pathway	KEGG_PATHWAY	8	12	11	2	2	4	-	-
IL-17 Family Signaling Pathways	SUPER_PATHWAY	9	2	4	-	-	-	-	-
Immune Response IL-23 Signaling Pathway	SUPER_PATHWAY	12	3	5	-	-	3	-	-
NOD-like Receptor Signaling Pathway	SUPER_PATHWAY	16	3	4	2	2	5	-	-
RIG-I-like Receptor Signaling Pathway	KEGG_PATHWAY	6	4	4	-	-	4	-	-
Inactivation of MAPK Activity	GO_MOLEC_FUNC	-	6	-	-	-	-	-	-
BAFF in B-Cell Signaling	SUPER_PATHWAY	4	9	8	-	-	-	-	-
Immune Response MIF-mediated Glucocorticoid Regulation	PATHWAY_MLPR	6	6	8	-	-	3	-	-
Immune Response IL-2 Activation and Signaling Pathway	SUPER_PATHWAY	7	9	7	-	-	-	-	-
**Compound/Immune Related**	** **	** **	** **	** **	** **	** **	** **	** **	** **
VEGF	COMPOUND	34	41	38	36	12	14	-	-
Rantes	COMPOUND	18	-	15	-	-	8	-	-
Nitric Oxide	COMPOUND	33	36	29	29	-	11	-	-
H2O2	COMPOUND	32	11	27	22	-	10	-	-
Progesterone	COMPOUND	33	10	-	25	14	8	-	-
Superoxide	COMPOUND	18	-	-	20	3	6	-	-
Histamine	COMPOUND	13	-	-	16	-	-	-	-
**Cellular/Signaling Related**	** **								
Homeostasis/Metabolism Phenotype	PHENOTYPE	107	100	80	74	37	24	-	-
Signal Transduction	GO_BIOL_PROC	51	-	24	30	-	13	-	-
PAK Pathway	SUPER_PATHWAY	37	-	19	-	-	-	-	-
Focal Adhesion	PATHWAT_KEGG	10	-	12	9	-	-	-	-
Cell Adhesion	GO_BIOL_PROC	20	19	-	18	-	-	-	-
p53 Signaling Pathway	SUPER_PATHWAY	7	-	10	-	-	-	-	-
Apoptosis Signaling	SUPER_PATHWAY	20	9	6	-	-	4	-	-
Integrin Pathway	SUPER_PATHWAY	15	20	17	-	-	-	-	-
EGFR1 Signaling Pathway	SUPER_PATHWAY	-	11	-	-	-	-	-	-
PI3K-Akt signaling Pathway	SUPER_PATHWAY	12	16	6	10	3	1	-	-

**Table 3:** Enrichment analysis using Set Distiller analysis from GeneDecks (version 3) showing descriptors/pathways and the number of genes modulated for virus, nervous and disorder related pathways. Enrichment analysis of pathways are statistically significant with a p-value <0.05 (Bonferroni corrected).

GeneDecks Set Distiller Analysis	Attribute Type	poly(I:C)	Lh-R0052	Bb-R0071	Bl-R0033	Lh-R0052-SLP	PC	PC	Lh-R0052-SLP
		only	poly(I:C)	poly(I:C)	poly(I:C)	poly(I:C)	poly(I:C)	only	only
**Virus Related**	** **	**Number of Genes Modulated**							
Influenza	DISORDER	11	27	-	8	-	-	-	-
Virus infection	DISORDER	24	20	20	-	-	8	-	-
Defense Response to Virus	GO_BIOL_PROC	13	-	-	-	-	-	-	-
Influenza A	SUPER_PATHWAY	20	15	-	-	-	-	-	-
Response to Virus	GO_BIOL_PROC	14	-	-	-	-	-	-	-
Type 1 Interferon Signaling Pathway	GO_BIOL_PROC	8	-	-	-	-	-	-	-
poly I:C	COMPOUND	8	8	6	-	-	-	-	-
Interferon-alpha	COMPOUND	7	-	-	-	-	-	-	-
2,5-oligoadenylate	COMPOUND	7	-	-	-	-	-	-	-
**Nervous/Endocrine Related**	** **	** **	** **	** **	** **	** **	** **	** **	** **
Nervous System Phenotype	PHENOTYPE	66	83	59	66	37	-	-	-
Behavior/Neurological Phenotype	PHENOTYPE	61	57	48	49	28	-	-	-
Endocrine/Exocrine Gland Phenotype	PHENOTYPE	47	55	33	37	15	12	-	-
**Disorder Related**	** **	** **	** **	** **	** **	** **	** **	** **	** **
Necrosis	DISORDER	70	67	56	53	20	23	-	-
Inflammation	DISORDER	60	53	49	40	21	22	-	-
Inflammatory Bowel Disease	DISORDER	15	26	20	10	9	-	-	-
Rheumatoid Arthritis	DISORDER	27	62	53	-	-	-	-	-
Multiple Sclerosis	DISORDER	15	39	35	-	8	-	-	-
Obesity	DISORDER	14	36	-	-	-	-	-	-
Autoimmune Disease	DISORDER	15	-	-	15	-	-	-	-
Gastritis	DISORDER	9	-	18	-	-	-	-	-

A secondary aim of this study was to evaluate other potentially interesting genes and go beyond the immune-related pathways that were previously reported in MacPherson *et al*. [22] by using genome-wide microarrays. Of particular interest was the capacity of Set Distiller enrichment to integrate gene function into disorder-related pathways. Examples included inflammation, inflammatory bowel disease, rheumatoid arthritis, multiple sclerosis, obesity and autoimmune diseases (Table 3). The enrichment analyses also revealed that there were a number of shared genes found in many of the disorder pathways (Table 3). The majority of these shared genes were associated with pro-inflammatory substances such as cytokines and chemokines that were also present in immune and inflammatory responses. Examples of such substances included chemokine (C-X-C motif) ligand 1 (*CXCL1)*, *CXCL2*, *CXCL3*, *CXCL10*, *CXCL11*, intercellular adhesion molecule 1 (*ICAM1*), interleukin 23 alpha subunit (*IL-23A*), *IL-17C*, *IL-26*, *IL-8*, interferon receptor 2 (*IFNAR2*), leukemia inhibitory factor (LIF) and tumor necrosis factor-alpha (*TNF-α*).

### Gene Interaction Network: String Analysis

**Fig 2:**
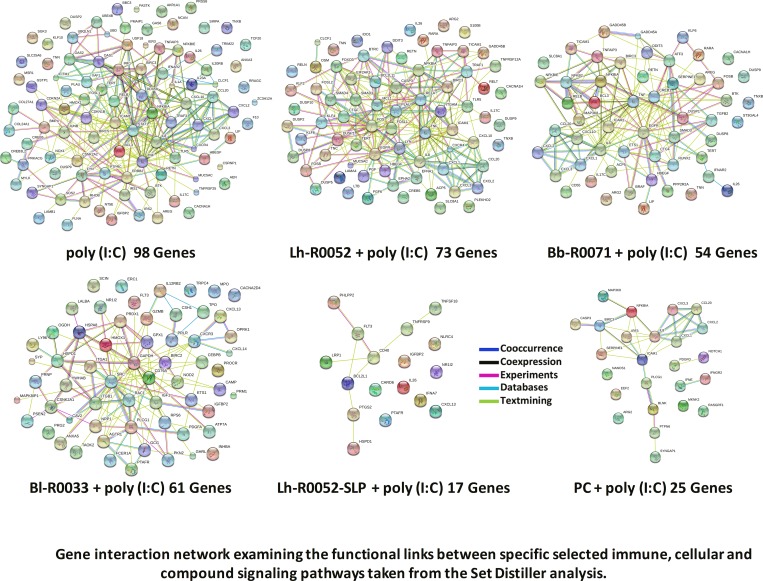
Gene interaction network map (String 9.1) examining the functional links between genes in specific immune, cellular and compound signaling pathways taken from the Set Distiller analysis. Selected pathways included apoptosis, NF-κB, MAPK, Jak-STAT, immune response IFN-alpha/beta, toll-like receptor, IL-17 family, immune response IL-23, RIG-I-like receptor, cytokine-cytokine receptor interaction, NOD-like signaling, nitric oxide, superoxide and histamine.

Enrichment was followed up for selected immune pathways, compound and cellular pathways to construct a gene interaction network map. String 9.1 was used to build this map and to determine functional links between genes. It was also used to confirm the Set Distiller analyses and elucidate potentially interesting functional links of genes that may have been previously overlooked. The String analysis was specified to look at the functional links of pathways involved in innate immunity and inflammation with a few cellular and compound pathways. This was done in order to make the functional link between innate immunity, inflammation, cellular signaling and compound to better elucidate, in part, the complex probiotic gene modulation that is exhibited by the single strains, multi-strain, PC, and Lh-R0052-SLP (Fig 2). It was evident from the analysis that there was, in fact, much overlap between genes in different pathway attributes. Many genes found in these respective linked pathways were also found in the disorders (Table 3) that are characterized by inflammation. The inflammation and inflammatory bowel disease (IBD) disorders had many commonly modulated genes that were also found in the String analysis that were common with the disorders which included *IL-8*, *ICAM1*, *RELB*, *NFκBIA*, *TNFAIP3*, *LIF*, *CXCL1*, *CXCL2*, *CXCL3*, *CXCL10*, and *TNF-α*.

One particular gene that stood out in the String analyses was *IL-17C* in the inflammation disorder (Table 3) that does not show up in the TNF, MAPK and NF-κB signaling pathways. In the Set Distiller analyses, *IL-17C* was only present in the immune system phenotype and IL-17 family signaling pathway (Table 2). When gene sets were analyzed with Set Distiller there was no other placement of this particular gene. Through the String analyses, *IL-17C* has functional links (Fig 2) to a key pro-inflammatory gene *TNF-α*, in turn having functional links to *CXCL1* and *IL-23A* (Fig 2), which are associated with the majority of disorder-related pathways. The use of complementary cross-enrichment analysis tools (Set Distiller and String) can be applied to extract useful functional insights or links of genes such as *IL-17C* and *IL-23A* that are important in the pro-inflammatory response of immunity and autoimmune or metabolic disorders. Another notable cytokine that was found was *IL-26*, which has a functional link to *IFNAR2*, in turn having a functional link with *IL-23A* (Fig 2). *IL-26* may also prove to be an important marker to monitor in immunity and inflammation as revealed in these results.

### Cytokine and Chemokine Profiling

**Fig 3:**
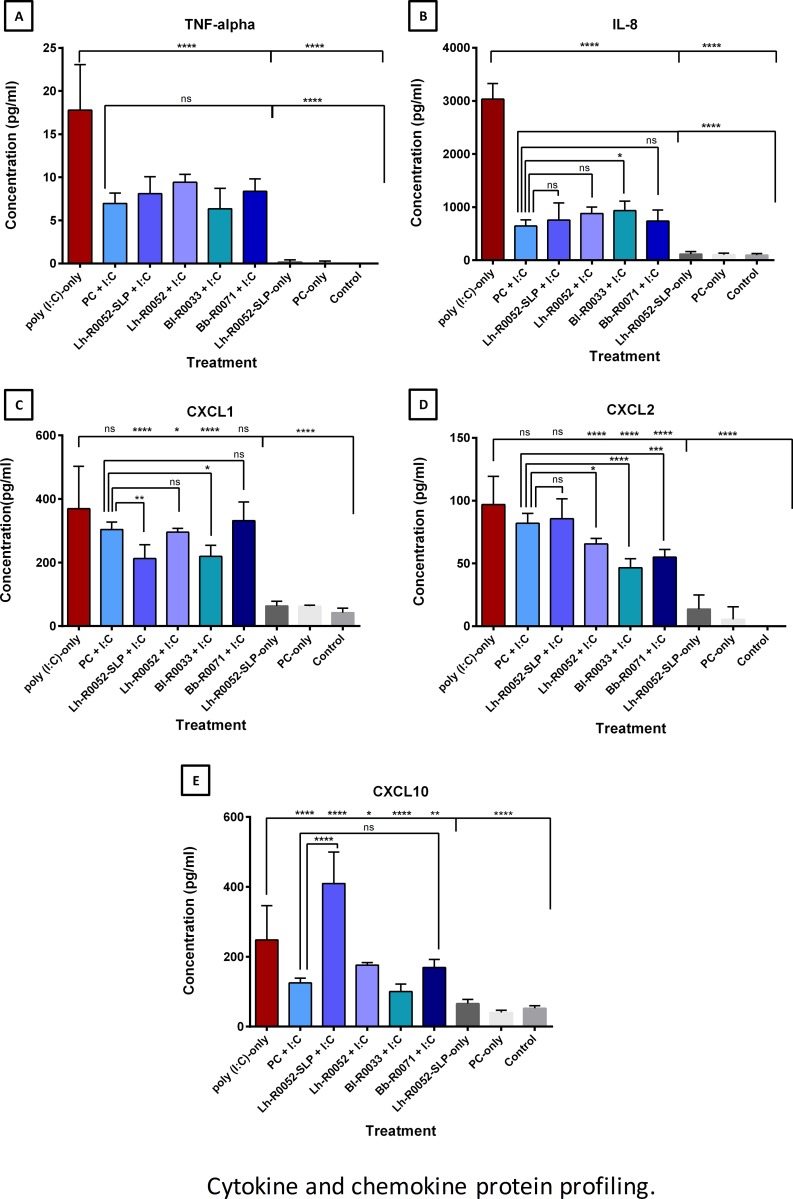
Cytokine and chemokine protein profiling for HT-29 cells challenges and co-challenges for (A) *TNF-α*, (B) *IL-8*, (C) *CXCL1*, (D) *CXCL2* and (E) *CXCL10*. Results were presented as the means ± SD of the replicate experiments (biological replicates n = 4; with 2 technical replicates each). One-way ANOVA using Dunnett’s multiple comparisons test was performed with GraphPad version 6 to determine the statistical significance with the pro-inflammatory stimulus, poly(I:C)-only and PC plus poly(I:C), *p*-values: *: *p*<0.05, **:*p*<0.01, ***: *p*<0.001, **** *p*<0.0001 and ns = not statistically significant.

Mapping out specifically where gene modulation lies in the TNF and NF-κB signaling pathway allowed a rational approach to selecting endpoints or markers to measure at the protein level. A core set of chemokines and cytokines were chosen as end points. This included *CXCL1*, *CXCL2*, *CXCL10* and *TNF-α* from the TNF pathway and IL-8 from the NF-κB signaling pathway. Protein levels of selected pro-inflammatory markers were analyzed using a fluorescent-magnetic-bead-based multiplex immunoassay. The results of Fig 3A and 3B for *TNF-α*, a key pro-inflammatory marker, and *IL-8*, a potent chemotactic factor that attracts neutrophils, basophils and T cells, showed a statistically significant (*p*<0.001) attenuation for each single strain, Lh-R0052-SLP and multi-strain PC co-challenges compared to poly(I:C)-only. Although there was more attenuation in the PC co-challenge for *TNF-α* and *IL-8* compared to the single strains and Lh-R0052-SLP, the difference was not statistically significant. However, for *IL-8* there was more attenuation for PC co-challenge compared to Bl-R0033 co-challenge (Fig 3B; *p*<0.05).

The chemokine markers *CXCL1*, *CXCL2* and *CXCL10*, that are involved in leukocyte recruitment, revealed strain, PC and Lh-R0052-SLP specific attenuation (Fig 3C, 3D and 3E). For example, PC and Bb-R0071 did not significantly attenuate *CXCL1* compared to the poly(I:C)-only; whereas the co-challenge of Lh-R0052-SLP (*p*<0.0001), Lh-R0052 (*p*<0.05) and Bl-R0033 (*p*<0.0001) did (Fig 3C). The results of *CXCL2* revealed that PC and Lh-R0052-SLP co-challenges did not significantly attenuate *CXCL2* protein levels compared to the poly(I:C)-only challenge; whereas Lh-R0052 (*p*<0.05), Bl-R0033 (*p*<0.001) and Bb-R0071 (*p*<0.001) co-challenges were all significantly attenuated (Fig 3D). All three single strains and the PC significantly attenuated *CXCL10* compared to poly(I:C)-only (Fig 3E), while Lh-R0052-SLP co-challenge significantly increased *CXCL10* levels compared to poly(I:C)-only. Protein measured for the negative control markers *IL-1β* and *IL-6* revealed no detectable protein modulation for all the challenges, confirming, as expected, the microarray expression data.

## Discussion

IEC act as a physical barrier and the first line of defense against pathogens and pathogen-associated molecular patterns (PAMPs) at the gut mucosal level and are an active participant in the host-microbiota cross-talk [31]. Recognition of potential threats and communication with the microbiota can occur via varied IEC signaling receptors [32]. Immune responses to PAMPs initiated at the IEC level activate immune cells, including macrophages, dendritic cells and B cells in the underlying gut associated-lymphoid tissues [33], leading to resolution of inflammation and the return to homeostasis. Intake of a specific strain or combination of bacteria and/or metabolic activity linked to these bacteria can attenuate TLR-activated innate immune responses by IEC [34]. However, few studies have established the efficacy of single strain versus multi-strain combinations on modulating TLR-induced inflammation in IEC. In this study, we utilized genome-wide microarray analysis to expand upon our previous findings with the custom-made immune array [22,27,35] and demonstrate distinct mechanistic differences at the expression level between single and the multi-strain probiotic combination on a broad range of immune and cellular pathways in poly(I:C)-challenged IEC.

The heat map and Set Distiller analyses revealed a number of key insights on the global impact of single strain (Bl-R0033, Lh-R0052 or Bb-R0071), probiotic combination (PC), and surface layer protein (Lh-R0052-SLP) on TLR-3 induced activation in IEC by poly(I:C). Both analyses reinforced that the strain specific and Lh-R0052-SLP effects differed from PC co-challenge. The effects of Lh-R0052 and Bb-R0071 diverged from that of Bl-R0033 and Lh-R0052-SLP, while PC modulation contrasted sharply with either grouping (Fig 1; Tables 2 and 3). Although the heat map analysis showed strain-based clustering, overall the effect of each probiotic treatment was quite different. It was evident from the global analyses with respect to the immune, cellular and homeostasis related pathways that there was a reduction in the total number of genes and pathways modulated by the single strains or Lh-R0052-SLP (Table 2). More specifically, the co-challenge with PC resulted in a substantial decrease in modulated genes and pathways, suggesting that the bacterial combination had a synergistic effect. Work performed by Chapman *et al*. [9] which investigated *in vitro* models of single and multi-strain probiotics on pathogen inhibition found that combinations had significantly greater inhibition of pathogens (e.g., *Clostridium difficile*, *Escherichia coli* and *Salmonella typhimurium*). The authors concluded that multi-strain formulations may be more effective at reducing gastrointestinal infections than single strain components [9]. Although the work by Chapman and co-workers used different *in vitro* models and pathogens, the results support the positive benefit of using multi-strain versus single strain preparations.

The enrichment analyses of the expression data using Set Distiller (Tables 2 and 3) in conjunction with String analysis (Fig 2) highlighted the overlap and functional connections between immune and cellular pathways. The findings demonstrated differences in the potential mechanisms by which single strain, Lh-R0052-SLP or PC co-challenge affected poly(I:C)-mediated inflammation in IEC. For example, the expression of *TLR5* and the mucin gene *MUC5AC* increased in poly(I:C)-challenged IEC and their expression was unaffected by the presence of Lh-R0052. Increased *TLR5* expression in TLR3-activated IEC is indicative of PRR cross-activation. The MYD88-TRIF adaptor cross-talk has been shown to synergize the immune response in the presence of TLR3 and TLR4 or TLR9 ligands and can act as a secondary signal to help the host evaluate an appropriate response to an immune challenge [36,37]. In contrast to Lh-R0052, Lh-R0052-SLP co-challenge increased the expression of the inflammasome component *NLRC4* (NLR family, CARD domain containing 4), demonstrating purified components from a probiotic strain can differ in their mechanism of action from whole bacteria.

The effect on gene expression in single strain Bl-R0033 co-challenged IEC was uniquely different from the other probiotic treatments. Firstly, Bl-R0033 up-regulated expression of genes *LY96 HSPA8* and *NOD2*, all of which assist with LPS-mediated inflammation. *LY96* encodes for a protein which associates with TLR4, while *HSPA8*, a heat-shock protein, binds to LPS [38]. *NOD-2* is a pattern recognition receptor found in the cytoplasm and recognizes peptidoglycans from both Gram positive and Gram negative bacteria [39]. Bifidobacteria can bind to LPS and while certain strains can inhibit LPS-induced NF-κB activation [40], this inhibitory effect is not universal across all strains [41]. However, as LPS was not used as a stimulant in the current study, the increased expression of LPS-associated genes indicates Bl-R0033 may precondition IEC to LPS presence during TLR3 activation.

In addition to the overlap in expression of genes involved in innate immune signaling, Bl-R0033 co-challenge also up-regulated *CD79*, which encodes for a B cell receptor component, and *CXCL13*, known to induce B cell homing to lymph nodes [38]. Although co-challenge with Bl-R0033 did not modulate B cell activating factor (*BAFF*) pathway genes, studies have shown *B*. *animalis lactis* Bb12 along with *L*. *rhamnosus* GG to secrete A Proliferation-Inducing Ligand (APRIL) from IEC [42]. APRIL and BAFF are cytokines that stimulate B cells to produce IgA in a T cell-independent manner [43]. Taken together, these findings suggest Bl-R0033 may, in TLR3-activated IEC, promote IEC-B cell communication at the gut mucosal level.

Genes involved in the attenuation of inflammatory signals were differentially expressed in single strain, PC and Lh-R0052-SLP co-challenge IEC. Both Lh-R0052 and Bb-R0071 co-challenge increased the expression of *DUSP9*, which encodes for a MAP kinase inhibitor, while Lh-R0052-SLP up-regulated *BCL2L11*, which inhibits *NLRC4* inflammasome activation [38]. Taverniti *et al*. showed that *L*. *helveticus* MIMLh5 surface-layer protein (SLP) induced anti-inflammatory effects by attenuating NF-κB activation in IEC, specifically Caco-2 cells [16]. These findings highlight that the effect of SLP is not only strain-specific, but that the possible mechanisms involved in dampening inflammation differ between probiotic components and whole bacteria.

The PC co-challenge gene expression profile demonstrated potential cross-inhibitory activity. The probiotic combination possibly attenuated TLR3-mediated inflammation in IEC by down regulating genes *IRF3* (downstream marker of TRIF), while up regulating genes *NF*κ*BIA* (inhibitor of NF-κB/REL complex) and *SYNGAP1* (inhibitory regulator of Ras-cAMP pathway) [38]. Multiple ligand engagement induces signals that cross-inhibit, leading to dampening of the inflammatory response [44] and may explain the PC effect on gene modulation. We also noted a significant decrease in protein levels of pro-inflammatory cytokine TNF-α and chemokines *IL-8* and *CXCL10* relative to poly(I:C)-challenged IEC and these possibly reflect PC-induced changes to the inflammation-associated gene expression profile. These findings agree with a previous preclinical assessment of the single strains and multi-strain combinations using two distinct rat models (T_H_1 and T_H_2) of infection which concluded that immune-modulation of T_H_1 and T_H_2 responses favored homeostatic rebalancing [11].

Further to attenuating inflammation, the PC co-challenge also modulated apoptosis-associated genes in our IEC model. Expression of *CASP3*, which initiates apoptosis, was down-regulated, while the gene *BIRC3*, an inhibitor of apoptosis, was up-regulated in PC co-challenged IEC. These genes were also modulated in the same manner in Lh-R0052 co-challenged IEC. In contrast, Lh-R0052-SLP down-regulated *CARD6*, which stimulates inflammasome-mediated apoptosis. Apoptosis can result from high levels of nitric oxide production [45] and TLR-mediated inflammation in IEC have been noted to be pro-oxidative, with TLR3 activation reducing the activity of super oxide dismutase [46]. However, the expression of the gene *ARG2* was down-regulated; indicating possible suppression of nitric oxide synthesis in Lh-R0052, Bb-R0071 and PC co-challenged IEC. In contrast, the expression of *PTAFR*, which is important for superoxide formation, increased in Bl-R0033 and Lh-R0052-SLP co-challenged IEC. Although Bl-R0033 co-challenge did not modulate the expression of genes in the apoptosis pathway, the increased expression of *PTFAR* highlights the complexity of the interaction of differing probiotic treatments at the cellular and immune level. Overall the functional link analysis (Fig 2) of immune and cellular gene pathways suggest that PC may attenuate TLR3-activated inflammation in IEC in a multi-pronged fashion, an approach that differed completely from single strains and Lh-R0052-SLP co-challenges.

The transcriptional analyses also revealed other cellular signaling, nervous and endocrine related disorders (Table 3). It has been widely reported in the literature that many cytokine and chemokine genes (e.g., *TNF-a*, *IL-8*, *CXCL2*, *CXCL3*, *CXCL10*, *LIF*, *IL-17C* and *IL-23A*) are the basis of autoimmune disorder pathways that are characterized by inflammation. For example, Yamaguchi *et al*. reported that *IL-17C* stimulates the production of *TNF-α*, in turn increasing inflammatory rheumatoid arthritis, and suggested that *IL-17C* may have a key role in the pathogenesis of arthritis [47]. Other reports have implicated *IL-17* in various autoimmune diseases such as multiple sclerosis and inflammatory bowel diseases [48]. In addition, other studies have also reported that *IL-17C* and *IL-23A* are key cytokines of T_H_17 responses that are involved in regulating innate immune functions and gut inflammation of IEC [49,50]. The fact that these particular cytokines were induced by the poly(I:C)-only challenge, and attenuated or turned off with the multi-strain PC, implies that these may be potential markers to follow in future probiotic clinical studies of these disorder-related diseases.

Overall the findings reveal a distinct pattern of gene modulation by single strains and Lh-R0052-SLP compared to the probiotic combination in TLR3-activated IEC. The genome-wide expression analysis demonstrated the probiotic combination modulated the least number of genes and through a highly regulated process attenuated TLR3-mediated inflammation in IEC, implying a synergistic effect at the expression level. Our findings highlight potential immune-modulatory mechanisms and biomarkers to follow in the rational design of future pre-clinical and clinical probiotic studies.

## Supporting Information

S1 Fig SDS-PAGE and 2D-gel analysis of purified Lh-R0052-SLP. (TIF)

S1 Table Gene expression data for selected pathways for String analysis in Fig 2. All genes were statistically significant with a p-value<0.05 and a cut-off fold change in transcript abundance of 1.5-fold.
(XLSX)

S2 Table Gene expression data for HT-29 cells for all challenges and co-challenges. All genes were significant with a p-value <0.05 and cut-off fold change in transcript abundance of 1.5-fold.
(XLSX)
